# Lean Mass and Musculoskeletal Preservation in GLP-1-Based Obesity Treatment: Nutrition, Exercise, Supplementation, and Monitoring Strategies

**DOI:** 10.3390/metabo16060364

**Published:** 2026-05-27

**Authors:** Roko Šantić, Lovre Martinović, Nikola Pavlović, Doris Rušić, Marko Kumrić, Dinko Martinović, Tina Tičinović Kurir, Joško Božić

**Affiliations:** 1 Department of Pathophysiology, University of Split School of Medicine, 21000 Split, Croatia; roko.santic@mefst.hr (R.Š.); lovre.martinovic@mefst.hr (L.M.); nikola.pavlovic@mefst.hr (N.P.); marko.kumric@mefst.hr (M.K.); tticinov@mefst.hr (T.T.K.); 2 Laboratory for Cardiometabolic Research, University of Split School of Medicine, Soltanska 2A, 21000 Split, Croatia; 3 Department of Pharmacy, University of Split School of Medicine, Soltanska 2A, 21000 Split, Croatia; doris.rusic@mefst.hr; 4 Department of Maxillofacial Surgery, University Hospital of Split, Spinciceva 1, 21000 Split, Croatia; dmartinovic@kbsplit.hr; 5 Department of Maxillofacial Surgery, University of Split School of Medicine, 21000 Split, Croatia; 6 Department of Endocrinology, Diabetes and Metabolic Diseases, University Hospital of Split, Spinciceva 1, 21000 Split, Croatia

**Keywords:** GLP-1 receptor agonists, semaglutide, tirzepatide, skeletal muscle mass, obesity, protein intake, dietary supplementation, bioelectrical impedance analysis

## Abstract

**Background/Objectives:** GLP-1-based obesity pharmacotherapy has shifted clinical attention from the magnitude of weight loss to the quality of weight loss. This review evaluates whether body composition changes during treatment with GLP-1-based agents represent clinically meaningful muscle loss and identifies nutrition, supplementation, exercise, and monitoring strategies that may help preserve lean mass, function, bone health, and nutritional adequacy. **Methods:** A comprehensive narrative review was performed using focused searches of PubMed, publisher-hosted journal platforms, and reference lists of key primary studies and recent evidence syntheses through March and May 2026. Evidence was organized around body composition, muscle quality and function, dietary protein and micronutrient adequacy, exercise, supplementation, bioelectrical impedance analysis, imaging, and emerging biomarkers. **Results:** Semaglutide and tirzepatide preferentially reduce fat mass, including visceral and ectopic adiposity, while producing smaller but consistent reductions in lean mass or lean soft tissue. However, DXA-derived lean mass and BIA-derived fat-free mass are not equivalent to skeletal muscle, and lean tissue loss does not necessarily indicate impaired strength or physical performance. The most defensible supportive care model combines food-first nutritional counseling, adequate protein intake, structured resistance exercise, management of gastrointestinal adverse effects, and risk-based monitoring of micronutrient inadequacy. Protein supplementation and nutritionally complete meal replacements may be useful when intake is insufficient, whereas creatine, essential amino acids or leucine, beta-hydroxy-beta-methylbutyrate, fiber, probiotics, omega-3 fatty acids, and multi-ingredient products remain adjunctive options supported mainly by indirect or phenotype-specific evidence. **Conclusions:** Future GLP-1 trials and clinical care should move beyond body weight and total lean mass toward integrated assessment of muscle quantity, muscle quality, function, bone, and nutritional adequacy, and standardized BIA-based clinical monitoring where advanced imaging is not feasible.

## 1. Introduction

The treatment landscape for obesity has changed rapidly. Once-weekly semaglutide 2.4 mg produced a mean body weight reduction of about 15% at 68 weeks in adults who were overweight or obese, tirzepatide extended pharmacological weight loss into the 15–21% range across phase 3 obesity trials, and longer-term maintenance studies have confirmed that continued therapy can sustain clinically meaningful reductions when treatment is not withdrawn [1,2,3,4,5]. These advances have repositioned obesity pharmacotherapy from modest adjunctive therapy toward a major pillar of chronic disease management [6]. As efficacy has increased, however, clinical discussion has shifted from kilograms lost to the composition and functional consequences of those losses.

Although obesity is a multisystem disease, the present review focuses specifically on how GLP-1-based weight loss affects musculoskeletal and nutritional health. Broader mechanisms, including adipokine signaling, metabolic flexibility, gut microbiota–metabolome interactions, and cardiometabolic physiology, are considered only where they influence body composition quality, nutritional adequacy, or functional outcomes [7,8,9,10].

This shift is justified. Weight reduction can improve glycemia, blood pressure, sleep apnea, mobility, liver fat, quality of life, and overall cardiometabolic risk, but the biological and clinical value of weight loss depends partly on what tissue is lost and what tissue is preserved [1,2,6,11]. In everyday discussion, terms such as “lean mass loss,” “muscle loss,” and even “sarcopenia” are often used interchangeably in the context of GLP-1 treatment. That is a conceptual mistake. A decline in dual-energy X-ray absorptiometry (DXA)-derived lean mass is expected during almost any substantial weight-loss intervention, including diet, lifestyle, bariatric surgery, and obesity pharmacotherapy, and it does not automatically indicate pathological skeletal muscle wasting [12,13,14,15]. The more relevant questions are whether lean tissue loss is proportionate to total weight loss, whether muscle quality and physical function deteriorate, whether bone is adversely affected, and whether reduced food intake leaves patients with inadequate protein, fiber, or micronutrients.

Several recent reviews, meta-analyses, and consensus statements have addressed body composition changes, nutritional priorities, or supportive care during GLP-1-based therapy [12,13,14,16,17]. However, the available literature remains fragmented. Body composition studies often report DXA-derived lean mass or lean soft tissue without parallel assessment of strength, physical performance, or nutritional adequacy. Nutrition, exercise, and supplementation recommendations are frequently extrapolated from the non-GLP-1 weight loss, older-adult, or sarcopenia literature, and biomarker-based approaches remain insufficiently prioritized for clinical practice. Therefore, a clinically oriented synthesis is needed to distinguish expected adaptive lean tissue remodeling from clinically relevant muscle deterioration and to clarify which supportive strategies are directly supported, indirectly supported, or still exploratory.

The answer is unlikely to be the same for every patient. Older adults, people with type 2 diabetes, individuals with low physical activity, those with pre-existing sarcopenic obesity, and patients who experience persistent nausea, vomiting, constipation, early satiety, or food aversion may all be more vulnerable to low-quality weight loss if supportive care is absent [18,19,20,21,22]. Conversely, patients who maintain adequate protein intake, preserve dietary variety, and engage in structured resistance exercise may lose substantial fat mass while stabilizing or improving muscle function and body composition quality [23,24,25,26]. The clinical challenge, therefore, is not to blunt the weight-loss effect of GLP-1 therapy, but to make that weight loss safer, metabolically richer, and more durable.

This review adopts a “quality of weight loss” framework. Within this framework, desirable treatment reduces fat mass, especially visceral and ectopic adiposity, while preserving the greatest possible amount of lean tissue, muscle quality, physical function, bone health, and nutritional adequacy. Unlike reviews that focus primarily on total lean mass change or isolated nutritional recommendations, this review integrates drug-specific body composition evidence with resistance exercise, protein adequacy, supplementation, imaging, functional assessment, biomarkers, bioelectrical impedance analysis (BIA) and phenotype-based clinical stratification. The intent is not to advance an alarmist narrative that GLP-1 therapies cause sarcopenia, but to provide a clinically useful, evidence-weighted roadmap for preserving musculoskeletal and nutritional health during highly effective obesity treatment [12,13,16,17].

## 2. Methods

This article was designed as a comprehensive narrative review rather than a systematic review or meta-analysis. The aim was to provide a clinically oriented synthesis of evidence on body composition changes, lean mass preservation, nutrition, exercise, supplementation, and monitoring strategies during GLP-1-based obesity pharmacotherapy.

A focused literature search was conducted through March and May 2026. PubMed/MEDLINE was used as the primary database, supplemented by targeted searches of Google Scholar, publisher-hosted journal platforms, and reference lists of key primary studies, systematic reviews, meta-analyses, consensus statements, and clinical guidance documents. Search terms included combinations of: “GLP-1 receptor agonist,” “glucagon-like peptide-1,” “semaglutide,” “liraglutide,” “tirzepatide,” “GIP/GLP-1,” “dual agonist,” “obesity pharmacotherapy,” “body composition,” “lean mass,” “lean soft tissue,” “fat-free mass,” “skeletal muscle,” “sarcopenia,” “sarcopenic obesity,” “muscle strength,” “physical function,” “DXA,” “MRI,” “CT,” “bioelectrical impedance analysis,” “BIA,” “bioimpedance,” “segmental bioelectrical impedance,” “multi-frequency bioelectrical impedance,” “phase angle,” “bioelectrical impedance vector analysis,” “extracellular water,” “extracellular water/total body water,” “protein intake,” “resistance exercise,” “resistance training,” “creatine,” “leucine,” “essential amino acids,” “HMB,” “omega-3 fatty acids,” “vitamin D,” “micronutrient deficiency,” “biomarkers,” “metabolomics,” and “proteomics.”

Eligible sources included randomized controlled trials, trial extension studies, imaging substudies, observational studies, systematic reviews, meta-analyses, expert consensus statements, and clinical nutrition or obesity-management guidance relevant to GLP-1-based therapy, body composition, skeletal muscle, nutritional adequacy, exercise, or supplementation. Direct evidence from GLP-1-treated populations was prioritized whenever available. When direct evidence was limited or absent, supporting evidence from non-GLP-1 weight-loss interventions, older-adult nutrition studies, sarcopenia research, resistance training studies, and the clinical nutrition literature was included if it was considered mechanistically or clinically relevant. Foundational papers on sarcopenia, sarcopenic obesity, body composition methodology, and protein requirements in older adults were also retained when needed to support definitions or interpretation. The final synthesis included 131 references; approximately 35–40 were primary GLP-1/GIP–GLP-1 clinical trials, extensions, imaging/body composition substudies, real-world cohorts, or mechanistic human studies, while the remainder consisted of non-GLP-1 supportive care studies, systematic reviews or meta-analyses, consensus or guidance documents, and methodological papers. When evidence conflicted, greater weight was given to human GLP-1/GIP–GLP-1 data with objective body composition, nutritional, or functional endpoints; indirect evidence was used mainly when direct evidence was unavailable and was interpreted according to clinical applicability, consistency, and outcome relevance.

Studies were excluded if they were not relevant to obesity pharmacotherapy, lean mass, muscle function, body composition, nutrition, exercise, supplementation, or biomarker-based monitoring. Animal or preclinical studies were not emphasized unless they provided mechanistic context not available from human studies. Opinion pieces, non-peer-reviewed sources, and commercial supplement claims were not used as primary evidence for clinical recommendations.

Because this was a narrative review, no PRISMA guidelines were used, no PRISMA flow diagram was generated, and the review was not registered in PROSPERO. No formal meta-analysis, pooled effect size calculation, or structured risk-of-bias assessment was performed. Instead, methodological limitations were considered narratively, with particular attention to study design, directness to GLP-1-treated populations, sample size, consistency of findings, outcome relevance, and translational applicability.

To improve transparency, supportive interventions were classified using a narrative strength-of-evidence approach. “Direct evidence” refers to studies conducted in GLP-1-receptor-agonist- or GIP/GLP-1-co-agonist-treated populations. “Indirect evidence” refers to evidence extrapolated from the obesity, caloric restriction, older-adult, sarcopenia, exercise, or clinical nutrition literature. Strength-of-evidence categories were assigned as follows: “moderate indirect” when consistent evidence existed from non-GLP-1 but clinically applicable populations; “limited direct” when GLP-1-specific data were available but sparse, observational, or not designed primarily around lean mass outcomes; “limited indirect” when evidence was heterogeneous, phenotype-specific, or extrapolated from related populations; and “very limited” when support was mainly mechanistic, exploratory, or lacked GLP-1-specific clinical validation.

## 3. Definitions, Measurement, and Clinical Relevance of Lean Mass, Muscle, and Function

### 3.1. Distinguishing Lean Mass, Fat-Free Mass, and Skeletal Muscle

The first barrier to a rigorous discussion of lean mass preservation is imprecise terminology. Lean body mass, fat-free mass, lean soft tissue, and skeletal muscle mass are related but distinct constructs [12,18,19]. DXA-derived lean mass includes water, organs, connective tissue, and other non-fat compartments in addition to skeletal muscle, and appendicular lean soft tissue remains only a proxy for actual contractile tissue. BIA-derived fat-free mass similarly estimates a non-fat compartment from electrical and hydration assumptions rather than directly measuring skeletal muscle. These distinctions matter in obesity trials because measured lean mass or fat-free mass can change without equivalent change in functional myofibrillar tissue [13,27,28,29,30,31,32,33,34]. Throughout the manuscript, terminology was standardized according to measurement method and biological specificity. “Lean mass” is used as a general umbrella term when discussing nonspecific non-fat tissue change; “lean soft tissue” is reserved for DXA-derived soft-tissue lean compartments; “fat-free mass” is used for BIA- or model-derived non-fat compartments; and “skeletal muscle mass” is used only when muscle-specific estimates, imaging-based muscle measures, D3-creatine dilution, or sarcopenia-related criteria are discussed. Study-specific terms are retained when reporting individual trials to avoid implying equivalence between these compartments.

The same conceptual caution applies to sarcopenia and sarcopenic obesity. Contemporary definitions emphasize a combination of low muscle quantity and impaired muscle strength or performance, rather than any numerical decline in lean mass or fat-free mass during treatment [18,19]. A patient may lose lean or fat-free mass during substantial weight reduction and still improve clinically if fat mass falls, mobility improves, and strength is maintained. Conversely, a patient with low baseline reserve may experience functional deterioration despite a smaller absolute tissue change.

### 3.2. Body Composition Assessment: BIA, DXA, MRI/CT, and D3-Creatine Dilution

Bioelectrical impedance analysis (BIA) estimates body composition from electrical impedance after passage of a low-level current through the body; resistance is influenced mainly by conductive body water, whereas reactance reflects capacitive properties related to cell membranes [31]. Single-frequency, multi-frequency, segmental, and spectroscopy-based BIA devices may estimate total body water, fat-free mass, fat mass, skeletal muscle mass, regional or appendicular compartments, hydration indices, and phase angle [32,33]. Its clinical strengths are portability, low cost, speed, repeatability, and absence of radiation, but outputs depend on device design, prediction equations, electrode configuration, posture, and hydration status [32,34].

DXA uses two X-ray energy levels to estimate fat mass, lean soft tissue, and bone density [35]. It provides whole-body and regional outputs, including appendicular lean soft tissue and bone mineral density, and has been used in major GLP-1 and GIP/GLP-1 body composition analyses [36,37]. However, DXA-derived lean soft tissue has non-contractile components and does not directly measure skeletal muscle quality, intramuscular fat, or neuromuscular function [12,13,35].

MRI and CT provide regional anatomic information on muscle volume, cross-sectional area, ectopic fat, and fat infiltration, and have been used in incretin studies to characterize liver fat, abdominal adiposity, and muscle composition [11,38,39,40]. MRI avoids ionizing radiation but is less scalable because of cost and access, whereas CT is limited for repeated monitoring by radiation exposure [38]. D3-creatine dilution estimates total body skeletal muscle mass from labeled creatine kinetics and urinary D3-creatinine enrichment, but it remains mainly a research method rather than a routine clinical tool in GLP-1-treated populations [41].

### 3.3. Functional and Skeletal Outcomes

Functional endpoints often matter more than static mass endpoints. Strength, physical performance, and cardiorespiratory fitness indicate whether tissue changes are clinically meaningful, and available incretin trial data show that modest lean mass decline can coexist with improved physical performance or handgrip strength [23,25,26]. Thus, a decline in DXA-derived lean mass or BIA-derived fat-free mass should not be interpreted as sarcopenia without functional context.

Bone deserves the same attention as muscle. Rapid weight loss, reduced mechanical loading, low calcium or vitamin D intake, and inadequate resistance exercise may threaten skeletal health, whereas exercise support during pharmacotherapy-assisted weight loss can help preserve bone mineral density [24]. Lean mass preservation should therefore be assessed together with strength, performance, bone health, and nutritional adequacy.

### 3.4. Framework for Evaluating the Quality of Weight Loss

Taken together, these measurement issues support a “quality of weight loss” framework. Successful GLP-1 treatment is characterized not merely by lower body weight, but by preferential fat loss, preserved or improved muscle quality and function, maintained bone health, and adequate nutrient intake throughout active treatment and maintenance [6,12,13]. BIA can contribute to this framework as a practical clinical monitoring tool, while DXA, MRI, CT, and functional testing help answer more specific questions about regional lean tissue, bone, muscle quality, and performance. This approach better matches the needs of clinicians deciding when to intensify nutritional counseling, involve dietitians, add resistance training, or investigate nutritional deficiency.

## 4. Body Composition Changes During GLP-1 Therapy: Current Human Evidence

### 4.1. Semaglutide

Semaglutide provides the most familiar modern evidence base for GLP-1-based obesity pharmacotherapy. In STEP 1, adults with overweight or obesity receiving semaglutide 2.4 mg once weekly lost about 15% of baseline body weight over 68 weeks compared with placebo, establishing a new benchmark for pharmacological weight reduction at the time [1]. After STEP 1, semaglutide efficacy was reproduced in complementary clinical settings, including adults with type 2 diabetes (STEP 2), weight-loss maintenance after run-in semaglutide exposure (STEP 4), longer-term follow-up over 104 weeks (STEP 5), and oral semaglutide obesity trials (OASIS 1 and later dose-ranging studies) [3,42,43,44,45]. From the perspective of lean mass preservation, however, efficacy alone is not the central question. The key issue is how that weight loss is partitioned across fat and lean compartments and whether composition changes are functionally acceptable.

The best-known semaglutide body composition data come from an exploratory DXA analysis of STEP 1. In that analysis, semaglutide reduced total fat mass by 19.3%, visceral fat mass by 27.4%, and lean body mass by 9.7%, while the proportion of body weight represented by lean body mass increased by 3.0 percentage points [36]. Because this was an exploratory analysis rather than a stand-alone primary endpoint paper, the findings should be interpreted cautiously. Even so, the direction of effect is consistent with broader experience: semaglutide produces substantial fat loss, including clinically important reductions in central adiposity, whereas absolute lean mass loss is smaller in proportional terms. Smaller clinical and real-world reports point in the same direction. In adults with obesity and type 2 diabetes, semaglutide improved phase angle and reduced fat mass, and in a smaller oral semaglutide study, fat mass decreased without a statistically significant reduction in muscle mass [46,47,48].

### 4.2. Tirzepatide

Tirzepatide has further intensified interest in body composition because its weight-loss efficacy is even greater. In SURMOUNT-1, adults with overweight or obesity lost roughly 15–21% of body weight at 72 weeks depending on dose [2]. Strong efficacy was also reproduced in SURMOUNT-2 among participants with type 2 diabetes and in later maintenance-style studies [4,5,49]. These larger absolute changes make tissue partitioning particularly important, because even a favorable ratio of fat-to-lean loss may translate into several kilograms of lean soft tissue loss in absolute terms.

The most informative direct body composition analysis comes from the DXA substudy of SURMOUNT-1. Look and colleagues reported that approximately three-quarters of total weight loss reflected fat mass and one-quarter reflected lean soft tissue; in absolute terms, tirzepatide-treated participants lost about 15.9 kg of fat mass and 5.6 kg of lean soft tissue over 72 weeks [37]. This partitioning is broadly consistent with other successful weight-loss interventions, although the absolute quantity of lean soft tissue lost may remain clinically relevant in older adults and other high-risk phenotypes. MRI data from SURPASS-3 provide a distinct tissue-level perspective: the original substudy demonstrated reductions in liver fat and abdominal adiposity [11], whereas the later post hoc analysis of the same MRI dataset reported lower muscle volume together with improved muscle fat infiltration [39]. These findings indicate that reductions in tissue volume do not necessarily imply deterioration in muscle composition.

### 4.3. Liraglutide

Although semaglutide and tirzepatide should dominate a contemporary review, earlier GLP-1 receptor agonist studies remain valuable because they provide more functional and behavioral detail than some newer obesity trials. The trial by Lundgren and colleagues examined weight-loss maintenance after an initial low-calorie diet and randomized participants to exercise, liraglutide, both, or placebo [23]. The study was not designed primarily as a lean mass trial, but it showed that combining pharmacotherapy with structured exercise produced better maintenance outcomes than either strategy alone. Later secondary analyses demonstrated clinically important preservation of bone and improvements in physical fitness when exercise was included, highlighting that the context in which weight loss is achieved matters as much as the medication itself [24,25].

Liraglutide studies also clarify why tissue quality must be assessed directly. In an MRI-based thigh muscle study, liraglutide reduced thigh muscle fat infiltration and adverse muscle composition despite reductions in body size and lower-limb tissue mass [40]. The study examined muscle fat infiltration and low muscle volume, not a separate adipose compartment between muscle groups, and should therefore be interpreted primarily in relation to muscle quality rather than total lean mass change [40].

### 4.4. Evidence from Meta-Analyses and Real-World Studies

Meta-analytic evidence supports a balanced interpretation. In a network meta-analysis of 22 randomized controlled trials, GLP-1 receptor agonists and co-agonists reduced total body weight, fat mass, and lean mass, but lean loss accounted for a minority of the overall weight change and relative lean mass generally did not deteriorate [14]. This body composition evidence is complemented by an earlier pooled analysis confirming clinically meaningful total weight loss across a broad range of GLP-1 receptor agonist trials, although that analysis did not formally quantify fat-to-lean partitioning [50]. Together, these findings argue against the claim that GLP-1 treatment produces a uniquely unfavorable form of tissue loss compared with other weight-loss strategies (summarized in Table 1).

Converging real-world evidence reinforces this interpretation, but cross-study nomenclature requires caution. The 2026 JAMA Network Open analysis used serial bioelectrical impedance analysis and therefore reported fat-free mass rather than DXA-derived lean mass or lean soft tissue. Both bariatric surgery and GLP-1 receptor agonist treatment reduced fat mass and fat-free mass, with a more favorable fat-mass-to-fat-free-mass reduction pattern after surgery; the proportion of total weight loss attributable to fat-free mass after GLP-1 receptor agonist treatment was approximately 29.8% at 6 months and 24.8% at 12 months [51]. These data do not support the proposition that GLP-1 therapy selectively depletes lean tissue beyond what is expected during substantial weight reduction. Experimental work integrating human and animal data reaches a similar conclusion [15]. Throughout this review, study-specific terms—lean body mass, lean soft tissue, or fat-free mass—are retained where measurement methods differ. Thus, the key quantitative signal across available studies is fat-dominant weight loss rather than selective lean tissue depletion; semaglutide reduced fat and visceral fat more than lean body mass, tirzepatide weight loss was approximately 74% fat mass and 26% lean soft tissue, and real-world BIA data suggest fat-free mass accounted for roughly one-quarter to one-third of total weight loss [36,37,51]. These values should be interpreted alongside function, because available trials show that lean mass or lean soft tissue reductions may coexist with improved fitness, handgrip strength, stair-climb performance, or bone outcomes when exercise and supportive care are included [23,24,25,26].

## 5. Translating GLP-1 Pharmacology into Tissue Remodeling

### 5.1. Shared Mechanisms of Lean Mass Change During Incretin-Based Obesity Treatment

Several shared mechanisms can explain why lean mass falls during successful GLP-1-based or dual-incretin obesity pharmacotherapy. The most obvious is a sustained energy deficit. Across available GLP-1 dietary data, reported reductions in energy intake range from approximately 16% to 39%, yet this caloric reduction is rarely accompanied by proportional dietary counseling [52]. Any intervention that reduces energy intake and body mass will reduce the mechanical and metabolic demands placed on the body, and some loss of fat-free tissue is therefore expected as part of adaptive remodeling [13,14,51]. This is not unique to incretin-based treatment. What matters is whether the reduction in lean tissue is disproportionate to total weight loss and whether it occurs alongside reduced strength, poor functional adaptation, or compromised nutritional status.

A second mechanism is reduced protein intake. Appetite suppression, early satiety, slower gastric emptying, nausea, vomiting, constipation, and taste or food preference changes can lower the absolute amount of food eaten and narrow the range of tolerated foods [21,22,53]. When total intake decreases without active counseling, protein intake can fall below levels needed to support muscle protein synthesis, particularly in older adults or in people who already consume little protein at baseline [16,52,54]. In practice, only 43% of current GLP-1 receptor agonist users achieve even the minimum protein target of 1.2 g/kg/day, and mean protein intake in one cohort was 77.3 g/day—well below estimated requirements [55].

A third mechanism is reduced mechanical loading. As body weight falls, daily activities impose less external load on skeletal muscle and bone. Unless resistance exercise or another adequate loading stimulus is introduced, the musculoskeletal system receives less reason to retain tissue [23,53,56,57]. This helps explain why exercise-containing interventions consistently outperform diet-only or pharmacotherapy-only approaches for preserving strength, function, and in some cases bone.

Finally, tissue remodeling during incretin-based treatment may be partly adaptive rather than harmful. Reduction in ectopic fat, intramuscular lipid, inflammation, and fluid or glycogen stores can lower measured lean mass while improving tissue quality and insulin sensitivity [15,39,40]. The clinical task is therefore to distinguish adaptive remodeling from avoidable depletion. That distinction cannot be made from body weight alone and is only partly captured by DXA. It requires attention to dietary intake, symptoms, baseline phenotype, functional testing, and eventually better biomarker-informed monitoring.

These mechanisms are amplified in vulnerable phenotypes. Older adults have greater anabolic resistance, lower habitual physical activity, and higher baseline risk of sarcopenia or osteopenia [19,58,59]. People with type 2 diabetes may begin treatment with lower muscle quality and greater ectopic fat, while those with sarcopenic obesity have less physiological reserve and more to lose if nutritional support is poor [18,39,49]. However, these shared downstream pathways do not mean that all agents acting on GLP-1-related pathways are pharmacologically interchangeable.

### 5.2. Agent-Specific Pharmacology and Body Composition Interpretation

These shared pathways should not be interpreted as evidence that liraglutide, semaglutide, and tirzepatide are pharmacologically interchangeable [12,13,14,15,60,61]. Liraglutide and semaglutide are GLP-1 receptor agonists with fatty acid acylation that prolongs systemic exposure through albumin binding, although liraglutide is administered once daily whereas semaglutide was developed for once-weekly administration [60]. Tirzepatide is mechanistically distinct because it is a dual GIP/GLP-1 receptor agonist with greater GIP receptor engagement and biased GLP-1 receptor signaling toward cAMP generation rather than β-arrestin recruitment [61]. These upstream pharmacological differences are clinically relevant because they may modify the magnitude, tempo, and tolerability of weight loss, thereby influencing the downstream lean tissue remodeling mechanisms described above rather than acting through a proven direct myotoxic effect [12,13,14,15,16,17,20,21,52,61]. Semaglutide represents a predominantly GLP-1-receptor-mediated model; in the STEP 1 body composition analysis, reductions in fat mass and visceral fat exceeded reductions in lean body mass, while the relative proportion of body weight represented by lean body mass increased [36,62]. Tirzepatide combines GIP and GLP-1 receptor activation and has produced greater weight and waist circumference reduction than semaglutide in direct comparison, but available SURMOUNT-1 and SURPASS-3 MRI data still indicate that lean soft tissue or muscle volume may decline while fat mass, ectopic fat, and muscle fat infiltration improve [2,11,37,39,61,63,64]. Liraglutide generally produces less weight loss than semaglutide in direct comparison, yet the liraglutide-plus-exercise literature remains important because it demonstrates that mechanical loading can modify musculoskeletal outcomes independently of pharmacological appetite suppression [23,24,25,40,65]. Therefore, agent-specific differences should be presented as modifiers of tissue remodeling rather than as evidence of uniform or inevitable lean mass depletion across GLP-1-targeting therapies [12,13,14,15,16,17,20,35,52].

### 5.3. GLP-1-Based Therapies Relative to Other Anti-Obesity Agents

Prior to GLP-1 receptor agonists, approved anti-obesity pharmacotherapy achieved modest and mechanistically narrow weight reduction [6,66]. Orlistat inhibits gastrointestinal lipase peripherally, reducing dietary fat absorption by ~30% and producing only 3–5% weight loss above placebo, with no effect on appetite, satiety signaling, or lean tissue [6,66,67]. Phentermine/topiramate suppresses appetite through adrenergic and GABAergic pathways, achieving 8–10% above placebo, the highest among older oral agents, but requires cardiovascular monitoring and is contraindicated in pregnancy [6,68]. Naltrexone/bupropion targets the hypothalamic opioid-dopaminergic reward pathway to reduce food intake, achieving approximately 4–5% above placebo, with neuropsychiatric monitoring requirements and contraindication in seizure disorders [6,66,69]. None of these agents were developed with a mechanistic rationale for lean mass preservation, and none carry dedicated nutritional or body composition monitoring frameworks [12,20,66].

Body composition data for older agents remain sparse. Orlistat’s weight loss is driven principally by fat malabsorption, while phentermine/topiramate and naltrexone/bupropion lack imaging-based body composition substudies, in contrast to GLP-1 therapies which have been rigorously characterized across more than 20 randomized controlled trials with DXA or MRI assessments [6,14,66,70]. Nutritionally, orlistat impairs fat-soluble vitamin absorption (A, D, E, K) with a well-established supplementation protocol, while older centrally acting agents do not compromise total dietary intake to a clinically significant degree and carry no dedicated nutritional monitoring requirements [16,20]. In contrast, contemporary GLP-1-based therapies can produce weight loss approaching bariatric surgery magnitudes, creating nutritional vulnerability that supports adapting structured protein and micronutrient monitoring principles from bariatric practice to GLP-1 care. [16,66,71,72].

## 6. Nutrition-Based Supportive Care to Preserve Lean Mass

### 6.1. Baseline Nutritional and Functional Assessment

Supportive care should begin with structured phenotyping rather than empiric supplementation. At present, no validated GLP-1-specific candidate selection score, nutritional risk score, or lean mass preservation algorithm has been endorsed by the joint advisory or Delphi consensus statements [16,17]. Standard eligibility for anti-obesity pharmacotherapy follows conventional obesity criteria, but supportive care intensity still relies on clinical judgment rather than a validated prediction tool [16,17,20,73]. This gap is not surprising; a 2026 systematic review found that only two of 41 randomized controlled trials prescribing liraglutide, semaglutide, or tirzepatide reported or assessed dietary change, and a 2025 scoping review found that only 10 of 129 injectable anti-obesity medication trials reported diet intake outcomes at all [74,75].

Accordingly, baseline assessment should document recent weight trajectory, protein intake, dietary variety, gastrointestinal symptoms, physical activity, strength or mobility, key comorbidities, renal function, and prior nutritional deficiency. When available, body composition measures can help identify low appendicular lean tissue or probable sarcopenic obesity, while phenotype-driven laboratory testing may include iron status, vitamin B12, folate, 25-hydroxyvitamin D, calcium-related measures, glucose control, and renal function, particularly in older adults or patients with restricted intake or prolonged gastrointestinal symptoms [76,77,78].

### 6.2. Protein as the Primary Nutritional Strategy

The rationale for prioritizing protein is stronger when the problem is quantified. In the cross-sectional study of current GLP-1 receptor agonist users, mean protein intake was 77.3 g/day, whereas estimated desirable intakes based on 1.2–2.0 g/kg/day corresponded to 74–169 g/day across participants; only 43% achieved at least 1.2 g/kg/day [55]. These observations are important because meta-analytic evidence from weight-loss interventions in adults with overweight or obesity indicates that higher protein intake attenuates loss of muscle mass, with intakes above about 1.3 g/kg/day associated with better preservation than lower intakes [79]. Current GLP-1 consensus documents therefore recommend individualized higher-protein dietary patterns, commonly around 1.2–1.5 g/kg actual body weight per day during active weight loss, while acknowledging that direct GLP-1-specific dose-finding evidence is still limited [17]. However, direct randomized evidence on protein supplementation during GLP-1 therapy remains absent; a registered trial is expected to provide initial data by 2027 [80].

Protein distribution also matters. In older adults, distributing protein across meals enhances the likelihood that each eating occasion reaches an anabolic threshold [58,59]. This point is particularly relevant during GLP-1 treatment, because low appetite commonly shifts intake toward a single large evening meal. In the secondary analysis of the same GLP-1 user cohort, diet quality was suboptimal and approximately 40% of daily protein was consumed at dinner [81]. A clinically rational approach is therefore to prioritize protein early in the day and at each eating occasion, using food-first strategies when possible and oral supplements when necessary.

Adherence to protein targets, however, cannot be assumed. GLP-1-induced appetite suppression, early satiety, nausea, and food aversion reduce total dietary intake, and without active dietary counseling protein intake is rarely prioritized spontaneously [20,21,55]. The finding that only 43% of current users achieve ≥1.2 g/kg/day, despite consensus guidance recommending this minimum, reflects not a lack of awareness but a structural difficulty inherent to the treatment [55]. Practical strategies to support adherence include using liquid or semi-solid protein sources during titration phases when solid food tolerance is reduced, incorporating protein into the first meal of the day rather than relying on evening intake, and engaging a registered dietitian to reinforce protein targets longitudinally rather than at a single consultation [16,17,55].

### 6.3. Meal Structure During Early Satiety and Gastrointestinal Adverse Effects

Meal structuring should be presented as a response to a documented problem, not as generic lifestyle advice. Available studies suggest that energy intake falls substantially during GLP-1 or dual GIP/GLP-1 therapy, typically by about 16–39%, but the composition of that reduced intake is rarely measured [52,74]. In current users, gastrointestinal symptoms are common, particularly nausea (53.7%), diarrhea (27.8%), and fatigue (30.3%) [55]. Under these conditions, otherwise appropriate advice on bulky vegetables, very high fiber loads, or large mixed meals may become impractical.

Practical dietary adaptation is therefore necessary. Smaller meals, slower eating, protein-first meal construction, temporary use of softer or less fatty foods during nausea, and separation of fluids from meals in patients with severe early satiety are pragmatic measures supported by current guidance [16,20,21,82]. Their purpose is to preserve adequate protein, energy, hydration, and dietary variety while symptoms are active.

### 6.4. Nutrient Density and Risk of Micronutrient Inadequacy During Low-Energy Intake

Reduced intake during GLP-1 therapy is not nutritionally neutral. In a 2025 cross-sectional study of GLP-1 receptor agonist users, mean daily intakes of several nutrients were frequently below Dietary Reference Intake (DRI) targets, including fiber (14.5 g/day; DRI 25–38 g/day), calcium (863 mg/day; DRI 1000–1200 mg/day), iron (12.1 mg/day; DRI 8–18 mg/day), magnesium (266 mg/day; DRI 310–420 mg/day), potassium (2186 mg/day; DRI 2600–3400 mg/day), choline (305 mg/day; DRI 425–550 mg/day), vitamin A (560 μg retinol activity equivalents/day; DRI 700–900 μg/day), vitamin C (51 mg/day; DRI 75–90 mg/day), vitamin D (4 μg/day; DRI 15 μg/day), and vitamin E (9.6 mg/day; DRI 15 mg/day) [55,83]. The same cohort also demonstrated poor adherence to several Healthy Eating Index components, including fruit, vegetables, whole grains, seafood and plant proteins, and fatty acid quality [81].

Clinical deficiency data point in the same direction. In a retrospective observational analysis of 461,382 adults with type 2 diabetes newly prescribed GLP-1 receptor agonists, diagnosed nutritional deficiencies occurred in 12.7% within 6 months and 22.4% within 12 months; vitamin D deficiency was most common, affecting 7.5% at 6 months and 13.6% at 12 months [76]. A food-first strategy remains the appropriate first-line response, but these data justify low thresholds for laboratory monitoring and correction of documented deficiency, especially in older adults, patients with restricted intake, and those with persistent gastrointestinal intolerance [73,77,78].

### 6.5. Role of Dietitian-Led Longitudinal Care

Longitudinal dietitian involvement remains underused despite these nutritional signals. In the 2025 cross-sectional study, only 20% of GLP-1 receptor agonist users reported referral to a registered dietitian nutritionist, and only 51% reported receiving information on management of gastrointestinal side effects [55]. This gap is consequential because nutritional priorities shift across treatment phases—from symptom control and hydration during titration, to muscle preservation and laboratory monitoring during active weight loss, to preventing diet quality deterioration and unresolved deficiency after treatment interruption [16,76]. The absence of a structured dietetic referral pathway for GLP-1 therapy stands in contrast to established post-bariatric practice, where comparable magnitudes of weight loss are managed with standardized, multidisciplinary longitudinal nutritional follow-up [73]. The clinical importance of this gap is reinforced by a 2026 systematic review and meta-analysis of 37 studies (*n* > 9300), in which West and colleagues reported that patients regained a mean of 0.4 kg per month after GLP-1 discontinuation, projecting toward baseline body weight within approximately 1.7 years, with full reversal of cardiometabolic gains within a similar timeframe. Notably, weight regain was faster after pharmacotherapy withdrawal than after cessation of behavioral interventions alone, indicating that pharmacotherapy without integrated nutritional and behavioral support produces a more fragile long-term outcome [84].

## 7. Exercise as a Co-Intervention for Lean Mass Preservation

### 7.1. The Insufficiency of General Physical Activity Guidance

Broad recommendations to “stay active” are unlikely to protect skeletal muscle during the magnitude of weight loss achievable with contemporary GLP-1 receptor agonists. Although aerobic activity confers meaningful cardiometabolic benefits and supports weight-loss maintenance, it does not supply the direct mechanical and anabolic stimulus that skeletal muscle and bone require to resist atrophy during energy deficit [53,56,57]. The mechanistic rationale is straightforward: pharmacologically driven weight loss reduces habitual gravitational loading, meaning that a deliberate, progressive loading stimulus becomes more important as body mass declines. Without it, the tissue-preserving potential of concurrent lifestyle behavior remains largely unrealized.

### 7.2. Resistance Training as Core Supportive Care

The most rigorously controlled randomized evidence in this field derives from the liraglutide–exercise maintenance program. Following an eight-week low-calorie-diet phase, participants were randomized to one year of placebo, liraglutide 3.0 mg/day, structured exercise, or their combination [23]. The exercise prescription required at least 150 min of moderate-intensity or 75 min of vigorous activity per week, incorporating two supervised sessions weekly alongside additional unsupervised sessions, with participants ultimately achieving approximately 116 min per week. Compared with pharmacotherapy alone, the combined arm produced an improvement in stair-climb performance approaching 9% and a meaningful increase in peak oxygen consumption expressed relative to fat-free mass [25]. Exercise-containing arms also preserved hip and lumbar spine bone mineral density substantially better than liraglutide alone, across sites of major clinical fracture risk [24]. Although these data derive from liraglutide rather than the newer, more potent agents, they constitute the most informative randomized evidence demonstrating that functional and skeletal outcomes require structured exercise support during pharmacologically induced weight loss.

Wider meta-analytic evidence from non-GLP-1 populations reinforces this conclusion, with the translational caveat that it derives from dietary rather than pharmacologically induced energy deficit. Across systematic reviews and meta-analyses in older adults and adults with obesity, resistance and combined training consistently attenuated fat-free mass loss during caloric restriction without impairing fat mass reductions, and produced more favorable lean mass outcomes than aerobic training alone, demonstrating that exercise modality matters when the clinical objective is preserving the compositional quality of weight loss [53,56,85,86]. Observational evidence further indicates that higher physical activity levels during intentional weight loss are independently associated with better fat-free muscle mass preservation, reinforcing the clinical relevance of structured loading across the weight-loss continuum [86].

Taken together, these analyses indicate that resistance training should not be viewed as interchangeable with other forms of exercise when lean mass preservation is the clinical objective, irrespective of how weight loss is induced. On this basis, resistance exercise should be integrated into the standard care framework for patients receiving GLP-1 receptor agonists, with a pragmatic minimum of two to three sessions per week targeting major muscle groups, progressed according to individual tolerance and functional capacity [57].

Despite consistent evidence supporting resistance exercise, real-world adherence remains a major limiting factor that clinical recommendations must acknowledge. Fatigue, GI adverse effects, and reduced energy availability, particularly during dose titration, can meaningfully reduce both exercise capacity and motivation [20,21,53]. In older adults and patients with type 2 diabetes, additional barriers include mobility limitations, fear of injury, peripheral neuropathy, and orthopedic comorbidities [18,19,57,58,59]. Where supervised programs are unavailable, a minimum effective dose framing of two sessions per week targeting major muscle groups is more feasible and more likely to be sustained than idealized protocols derived from the sports science literature [53,56,57]. Resistance exercise recommendations should therefore be individualized, phase-sensitive, and accompanied by realistic expectations about what is achievable during dose escalation versus maintenance.

### 7.3. Aerobic Training, Habitual Movement, and Skeletal Health

Aerobic exercise and habitual physical activity retain a meaningful complementary role alongside resistance training during GLP-1 receptor agonist therapy, improving cardiorespiratory fitness, insulin sensitivity, and long-term weight-loss maintenance irrespective of body composition effects [56]. However, aerobic training alone cannot be assumed to preserve fat-free mass under conditions of marked caloric restriction; a systematic review and meta-analysis found its protective effect substantially weaker than that of resistance or combined modalities [87]. A critical limitation applies across this entire evidence base: virtually all available studies have examined dietary- rather than pharmacologically induced weight loss, and GLP-1 receptor agonist therapy, characterized by sustained appetite suppression, altered gut hormone signaling, and rates of weight loss exceeding what dietary restriction typically achieves, represents a physiologically distinct environment into which these findings cannot be directly extrapolated [24]. The available evidence should therefore be interpreted as establishing mechanistic plausibility rather than direct proof of efficacy in the GLP-1 context.

This caveat also applies to skeletal health. In dietary restriction models, resistance or combined exercise generally better preserves bone mineral density during weight loss than aerobic training alone, particularly at the hip and femoral neck. [88]. The only randomized trial to have directly assessed these outcomes under GLP-1 receptor agonist conditions, the secondary bone analysis of the liraglutide–exercise maintenance program, found that liraglutide alone reduced bone mineral density at the hip and lumbar spine to a greater degree than exercise alone despite comparable weight loss, whereas exercise-containing arms preserved bone mineral density at the hip, lumbar spine, and distal forearm, with the combination producing the greatest overall skeletal protection [24]. The authors noted that the magnitude of weight loss in their trial was comparable to that achievable with contemporary semaglutide and tirzepatide regimens, lending clinical relevance to findings generated with an earlier agent [24]. Nevertheless, this remains the only randomized evidence of its kind, and whether the same protective effects of exercise will be observed under the greater and more sustained pharmacological suppression produced by newer agents has not yet been established. There is also a growing concern that GLP-1 receptor agonist therapy may influence bone metabolism independently of weight loss, through direct receptor-mediated effects on bone remodeling, introducing a variable entirely absent from diet-only models that may both amplify skeletal risk and alter the exercise response in ways not yet fully characterized [89].

Framing physical activity as a continuum, from habitual daily movement through structured aerobic and resistance training, rather than as a binary exercise-or-not decision is the approach most consistent with the current evidence base, while acknowledging that most of the supporting data come from dietary restriction contexts and that the full exercise response profile specific to the GLP-1 treatment era remains to be defined by dedicated trials [57].

## 8. Supplementation Strategies During GLP-1 Therapy

### 8.1. Oral Protein Supplementation and Meal Replacements

Supplementation decisions during GLP-1 receptor agonist therapy should follow clinical indication rather than market trends, and within this hierarchy, oral protein supplements and nutritionally complete meal replacements occupy the most defensible position. Their rationale is straightforward: they directly address the documented shortfall between actual and target protein intake in this population. Cross-sectional data confirm that the majority of current GLP-1 users fail to reach the 1.2–2.0 g/kg/day intake considered appropriate during pharmacologically induced hypocaloric weight loss, a gap compounded by persistent gastrointestinal side effects that further narrow dietary variety and meal volume [21,22,55].

The clinical significance of closing this gap is well-supported; meta-analytic evidence from 47 randomized trials confirms that enhanced protein intake attenuates lean mass loss, with intakes above 1.3 g/kg/day associated with greater muscular preservation [79]. Multiple consensus and clinical guidance documents converge on the same framework, that food-first nutritional counseling should form the foundation of care, with supplementation reserved for patients unable to meet targets through diet alone, and with protein intake targets explicitly linked to concurrent resistance training to fully realize their lean mass benefits [16,17,54,90,91,92,93].

Product selection should remain pragmatic because GLP-1-specific data do not identify superior formulations or dosing schedules. Indirect evidence suggests that protein supplementation has more consistent lean mass benefits when combined with structured resistance exercise than when used alone [94]. This exercise dependency reinforces a central theme of this review, that nutritional and exercise co-interventions are interdependent rather than interchangeable.

Where additional anabolic potency is sought, particularly in higher-risk patients such as older adults or those with sarcopenic obesity, enriched formulations show greater promise. A randomized controlled trial in obese older adults found that a supplement combining whey protein, leucine, and vitamin D preserved significantly more appendicular lean mass compared with an isocaloric control during a 13-week hypocaloric weight-loss program, without attenuating fat loss [95]. The utility of novel protein sources such as plant-based or yeast-derived proteins is less established; a 6-month randomized trial comparing whey with yeast protein supplementation in elderly adults found favorable effects on muscle mass, strength, and function from whey, while yeast protein produced more modest and less interpretable results [96]. Until a richer GLP-1-specific evidence base emerges, product choice should be guided by tolerated volume, protein dose per serving, leucine content, micronutrient composition, lactose tolerance, and renal status rather than commercial claims [54,91,92,94].

### 8.2. Micronutrient Supplementation in Patients at Risk of Inadequate Intake

Micronutrient supplementation during GLP-1 receptor agonist therapy should remain phenotype-driven rather than reflexive. The current evidence does not support universal multicomponent regimens specifically marketed for GLP-1 users; instead, risk-stratified intervention is warranted when oral intake is persistently restricted, gastrointestinal symptoms are prolonged, or biochemical evidence of deficiency is present [76,77,91,92]. Within this risk-based framework, not all micronutrients carry equal priority. Vitamin D and calcium are most relevant when skeletal risk or documented inadequate intake is present, whereas iron, vitamin B12, folate, and thiamine deserve particular attention in patients with prolonged low oral intake, recurrent vomiting, or marked dietary monotony, patterns that progressively narrow both food variety and nutrient density, conditions under which absorption of water-soluble vitamins and non-haem iron is most vulnerable to cumulative depletion. [76,77].

A useful conceptual anchor for structuring this monitoring approach comes from the bariatric surgery literature, where dietary restriction and altered gastrointestinal physiology create a comparable profile of nutritional vulnerability. That experience argues for structured, interval-based monitoring of micronutrient status calibrated to symptom burden and individual intake trajectory, rather than empiric supplementation applied uniformly across all patients [78]. Translating this principle to the GLP-1 context, multivitamin–mineral preparations may be reasonable when food variety has narrowed substantially, but they should complement rather than substitute for biochemical monitoring and individualized dietetic assessment, a distinction that becomes especially important in older adults, those with comorbidities, and patients experiencing persistent gastrointestinal symptoms [54,77,78,91,92].

### 8.3. Creatine Monohydrate

Among muscle-oriented adjuncts, creatine monohydrate carries one of the stronger indirect evidence bases available, and its biological rationale translates plausibly to the GLP-1 treatment. Phosphocreatine stores in skeletal muscle replenish adenosine triphosphate during high-intensity effort, support satellite cell activity, reduce markers of oxidative stress, and may upregulate anabolic signaling pathways that sustain lean tissue during periods of energetic stress [97]. These mechanisms are not unique to athletic populations; they are directly relevant to patients undergoing pharmacologically induced weight loss, in whom reduced habitual loading and hypocaloric intake simultaneously suppress the anabolic environment that muscle requires to be maintained.

The external evidence in older adults is the most applicable evidence available. Two independent 2025 meta-analyses of older adults undergoing resistance training found that creatine supplementation produced modest but consistent improvements in lean tissue mass and muscular strength; one reported significant gains in one-repetition-maximum strength, and the other identified larger lean tissue effects in interventions of 32 weeks or less [98,99]. Although not GLP-1-specific, available creatine evidence supports biological plausibility and selective clinical consideration in patients performing resistance training, while direct efficacy data in GLP-1-treated cohorts remain absent. [54]. At the practical level, a maintenance dose of approximately 3–5 g/day of creatine monohydrate taken daily, including rest days, is consistent with current usage in the aging and resistance training literature and carries a well-established safety profile across the relevant patient phenotypes [97].

### 8.4. Leucine, Essential Amino Acids, and Beta-Hydroxy-Beta-Methylbutyrate

Leucine-enriched formulations, essential amino acid mixtures, and beta-hydroxy-beta-methylbutyrate (HMB) target muscle protein synthesis and anti-proteolytic pathways more directly than general protein fortification, and their proposed utility during GLP-1 therapy derives from the same mechanistic logic applicable during other forms of caloric restriction [100]. A 2025 meta-analysis of 21 randomized trials in adults over 50 years of age found that HMB supplementation was associated with modest but statistically significant improvements across multiple endpoints, appendicular skeletal muscle mass, lean mass, handgrip strength, gait speed and chair-stand performance, with subgroup analyses suggesting more pronounced effects when combined with exercise [101]. An international position stand on HMB similarly characterizes the compound as a biologically plausible anti-catabolic agent, while cautioning that effect sizes remain modest and population-specific, and that evidence in obesity and metabolic disease contexts is limited relative to the broader sarcopenia literature [102]. No direct randomized evidence yet demonstrates that any of these amino-acid-based strategies specifically attenuate lean tissue decline during semaglutide or tirzepatide treatment, and they should be framed as adjunctive and hypothesis-generating options rather than established standards of care [90,100].

### 8.5. Fiber, Probiotics, Omega-3 Fatty Acids, and Multi-Ingredient Formulations

A second cluster of adjuncts targets treatment tolerability and the broader nutritional environment rather than direct anabolism, and for this reason their evidence base is evaluated differently from the protein and creatine literature reviewed above. Soluble and prebiotic fiber preparations represent the most immediately practical option within this group, potentially helping to manage constipation and partially offset the low fiber intakes documented in current GLP-1 users; however, tolerance must be assessed individually, because excessive bulking can aggravate the fullness, bloating, and nausea that are already prevalent during treatment initiation and dose escalation [22,55,82]. Probiotic and synbiotic strategies are even less developed in this specific context and are better framed as gastrointestinal support than as evidence-based lean mass interventions; their clinical utility during GLP-1 therapy remains exploratory within current consensus frameworks, and the mechanistic pathway from gut microbiome modulation to meaningful muscle preservation has not yet been established in this population [91,92].

Omega-3 fatty acids occupy a different position in this hierarchy, one characterized by a stronger mechanistic rationale but persistent clinical inconsistency. Eicosapentaenoic acid and docosahexaenoic acid are incorporated into skeletal muscle membrane phospholipids, where they may modulate membrane fluidity, inflammatory lipid mediator production, anabolic signaling, and muscle protein turnover, a mechanistic profile that provides genuine plausibility for lean mass protection during hypocaloric states [103]. Yet translating these mechanisms into consistent clinical outcomes has proven difficult. A 2025 overview synthesizing 33 systematic reviews found sufficient evidence for benefit in only a minority of reviews examining lean mass, strength, and physical function outcomes, leading the authors to conclude that omega-3 supplementation cannot currently be recommended for lean mass preservation on the available evidence [104]. This gap between biological plausibility and clinical demonstration is a recurring theme across the adjunct literature and reinforces the importance of distinguishing mechanistic promise from proven efficacy.

Multi-ingredient formulations that combine high-quality protein with creatine, vitamin D, calcium, or omega-3 fatty acids represent a logical next step beyond single-ingredient strategies, and combinatorial approaches are increasingly supported by the geriatric and sarcopenia literature as more likely to address the multifactorial nature of muscle loss in older and clinically complex patients [95,105]. Nevertheless, direct evidence in GLP-1-treated cohorts remains absent, and the leap from sarcopenia trial data to pharmacologically induced weight loss requires the same translational caution applied throughout this review. Until dedicated trials in GLP-1-treated populations are completed, multi-ingredient supplementation should be viewed as a hypothesis-generating strategy with biological plausibility rather than an evidence-based standard of care (Table 2).

## 9. Biomarkers and Multimodal Phenotyping for Lean Mass Preservation

### 9.1. Rationale for Biomarker Integration

The rationale for biomarker integration is methodological rather than speculative. Body weight, fat mass, and DXA-derived lean tissue describe the magnitude of change, but they do not determine whether an individual patient is undergoing favorable fat-dominant remodeling, nutritionally poor adaptation, inflammatory stress, or early functional compromise. Biomarkers become relevant only if they improve phenotyping, risk stratification, or trial enrichment beyond what can be obtained from imaging and performance testing alone [106,107,108,109]. The biomarker landscape relevant to lean mass preservation during GLP-1 therapy spans from established clinical monitoring tools to early-stage research instruments, and these categories carry fundamentally different implications for practice.

### 9.2. Body Composition Monitoring During GLP-1-Based Therapy

BIA is best positioned as a low-burden longitudinal monitoring tool when information beyond body weight and waist circumference is needed during GLP-1-based therapy [31,32,34]. Direct GLP-1/BIA evidence remains mostly observational, but semaglutide, oral semaglutide, tirzepatide, and real-world GLP-1 receptor agonist cohorts have used BIA to track fat mass, fat-free mass, skeletal muscle estimates, visceral adiposity, body water indices, or phase angle [46,47,51,110,111,112,113]. BIA is most useful in patients with rapid weight loss, poor intake, persistent gastrointestinal symptoms, older age, type 2 diabetes, suspected sarcopenic obesity, or declining strength or mobility [18,19,21,76]. Results should be interpreted as trends under comparable testing conditions, because hydration, device type, equations, and measurement setup can alter fat-free mass estimates [31,32,34,114,115]. Falling fat-free mass or phase angle should prompt nutritional and functional reassessment rather than automatic labeling as sarcopenia [18,19,116,117].

DXA should be considered when appendicular lean soft tissue, regional fat-to-lean partitioning, or bone mineral density would change management [24,35,36,37]. It is particularly relevant in older adults, postmenopausal women, patients with fracture risk, suspected sarcopenic obesity, or unexplained functional decline [18,19,24]. In trials, DXA provides more standardized regional lean tissue and bone outcomes than BIA, but DXA-derived lean soft tissue still requires interpretation alongside strength and performance measures [12,13,35].

MRI or CT should be reserved mainly for research or selected high-risk cases requiring more precise assessment of muscle quality, myosteatosis, ectopic fat, or muscle volume [11,38,39,40]. Opportunistic CT may be informative when scans are already obtained for clinical indications, but CT should not be used solely for frequent routine monitoring because of radiation exposure [38]. D3-creatine dilution is a research-priority method for future GLP-1 studies in older adults and sarcopenic obesity because it estimates total body skeletal muscle mass more directly than lean tissue proxies, but it is not yet routine clinical monitoring in this setting [41].

### 9.3. Circulating Biomarkers of Muscle, Inflammation, and Nutritional Adequacy

Among circulating biomarkers, creatinine–cystatin C-based indices are currently among the most scalable options because they can be derived from routine laboratory data. Their limitation is that they remain only moderately accurate. In a 2024 diagnostic meta-analysis, pooled sensitivity and specificity for sarcopenia were 65% and 79%, respectively [118]. These indices may therefore be useful for screening or risk enrichment, but not as stand-alone outcome measures. Conventional inflammatory markers such as C-reactive protein, interleukin-6 (IL-6), and tumor necrosis factor-alpha (TNF-alpha) are even less specific. They may reflect catabolic or inflammatory burden, but they do not distinguish muscle loss from broader systemic illness [108,109,119,120].

Other candidates are biologically attractive but clinically immature. Myostatin is closely linked to muscle biology, yet observational data remain inconsistent and context dependent [121]. Notably, a 2026 blinded crossover proteomics study reported that short-term liraglutide substantially modulated 124 proteins and uniquely suppressed myostatin-related signaling compared with placebo [122]. However, an exploratory 18-day liraglutide crossover study found reductions in body mass and regional tissue mass without clear changes in the myostatin-activin-follistatin-insulin-like growth factor-1 (IGF-1) axis [123]. Growth differentiation factor-15 is better interpreted as a broader signal of frailty, symptom burden, and catabolic stress than as a muscle-specific marker [124]. Routine nutritional biomarkers, ferritin, transferrin saturation, vitamin B12, folate, thiamine, and 25-hydroxyvitamin D remain the most clinically actionable markers because they help separate favorable tissue remodeling from nutritionally poor adaptation [76,77,78,109,119].

### 9.4. Omics and Pharmacogenomic Approaches

Metabolomics, lipidomics, and proteomics extend this framework from single analytes to pathway-level phenotyping. In obesity treated with liraglutide, plasma metabolomic profiling demonstrated 161 significantly altered metabolites after 12 weeks, including oxylipins, bile acids, N-acylated amino acids, and metabolites linked to inflammatory and oxidative pathways [106]. Semaglutide proteomics similarly identified broad changes in cardiometabolic, inflammatory, and extracellular matrix pathways [107]. Such datasets remain exploratory, but they are valuable because they may distinguish favorable adiposity reduction from nutritionally compromised or catabolic adaptation that is not evident from body weight alone.

Equivalent omics data for tirzepatide in humans remain limited. Current tirzepatide-specific mechanistic evidence is weighted more toward imaging and whole-body physiology than toward metabolomics or proteomics. In a 2025 human mechanistic trial, tirzepatide increased fat oxidation and reduced ad libitum energy intake but did not attenuate the metabolic adaptation that commonly accompanies weight loss [64]. In parallel, pharmacogenomic work has begun to identify treatment response variability; a 2026 genome-wide analysis of nearly 28,000 GLP-1 receptor agonist users identified glucagon-like peptide-1 receptor (GLP1R) and glucose-dependent insulinotropic polypeptide receptor (GIPR) variants associated with differences in efficacy and gastrointestinal adverse effects, with GIPR-related signals particularly relevant to tirzepatide exposure [125].

### 9.5. Implications for Clinical Trial Design and Current Limitations

For clinical trial design, biomarkers and body composition tools should complement, not replace, structural and functional endpoints. DXA is appropriate for serial estimation of total fat mass, appendicular lean soft tissue, and bone mineral density, whereas MRI or CT is required when the objective is to characterize muscle volume, myosteatosis, or other aspects of muscle quality [35,38,39]. BIA may be useful when repeated DXA or MRI is impractical, but studies should report the device, prediction equation, and measurement conditions, and should clearly distinguish BIA-derived fat-free mass from DXA- or MRI-derived outcomes [28,29,30,31]. Functional measures such as handgrip strength, chair-rise time, gait speed, stair-climb performance, 6 min walk distance, and cardiorespiratory fitness remain necessary to determine whether tissue changes are clinically relevant [23,24,25,26]. Laboratory panels and omics platforms may improve risk stratification and responder phenotyping, but the field still lacks standardized panels, validated thresholds, and prospective evidence that biomarker-guided supportive care improves outcomes [35,107,108,119].

Accordingly, the most defensible near-term model is multimodal rather than biomarker-centric: structured dietary assessment, targeted laboratory monitoring, standardized BIA where available, functional testing, and DXA or MRI/CT in selected patients, with omics and pharmacogenomics incorporated primarily in research settings. Such an approach is more consistent with current evidence than presenting any single body composition output, metabolite, cytokine, or omics profile as a clinically actionable measure of lean mass preservation. At present, metabolomic, proteomic, lipidomic, and pharmacogenomic profiles should therefore be considered research tools for mechanism discovery, responder phenotyping, and trial enrichment, not validated instruments for selecting individualized protein, exercise, supplement, or dose-adjustment strategies in routine GLP-1 care [106,107,122,125]. In clinical practice, biomarker use should remain anchored in routine nutritional and safety laboratories, symptom burden, dietary intake, standardized body composition trends, and functional assessment.

## 10. Population-Specific Considerations and Treatment Phases

### 10.1. Older Adults

Older adults warrant the highest level of caution because they begin treatment with lower anabolic reserve, higher baseline prevalence of mobility impairment, and greater risk of osteopenia or sarcopenia [18,19,59]. In these patients, the objective is not weight reduction alone, but preferential fat loss with preservation of strength, function, and skeletal health. Practical implications include earlier dietetic involvement, lower thresholds for resistance exercise referral, and more frequent reassessment of symptoms, intake, and functional performance [57,58].

### 10.2. Sarcopenic Obesity

Individuals with sarcopenic obesity represent a phenotypically distinct high-risk group in whom excess adiposity coexists with low muscle mass, reduced muscle quality, or impaired physical function [18,19]. This combination reduces physiological reserve and amplifies vulnerability to nutritionally poor adaptation during GLP-1-based weight loss, particularly when supportive care is absent [12,13,20,76]. Clinical management should therefore prioritize preservation of lean tissue and functional capacity alongside fat reduction, with structured resistance exercise and adequate protein intake as core co-interventions rather than optional adjuncts [16,53,57,90,126].

### 10.3. Type 2 Diabetes and Lower Functional Reserve

Patients with type 2 diabetes may also require closer monitoring because baseline muscle quality, ectopic fat burden, neuropathy, and cardiometabolic comorbidity are often less favorable than in obesity cohorts without diabetes [39,42,49]. Improvements in liver fat and visceral adiposity may be substantial, but glycemic response should not be assumed to mirror preservation of fat-free tissue, nutritional adequacy, or function.

### 10.4. Treatment Phases: Initiation, Active Loss, Maintenance, and Withdrawal

**Figure 1:**
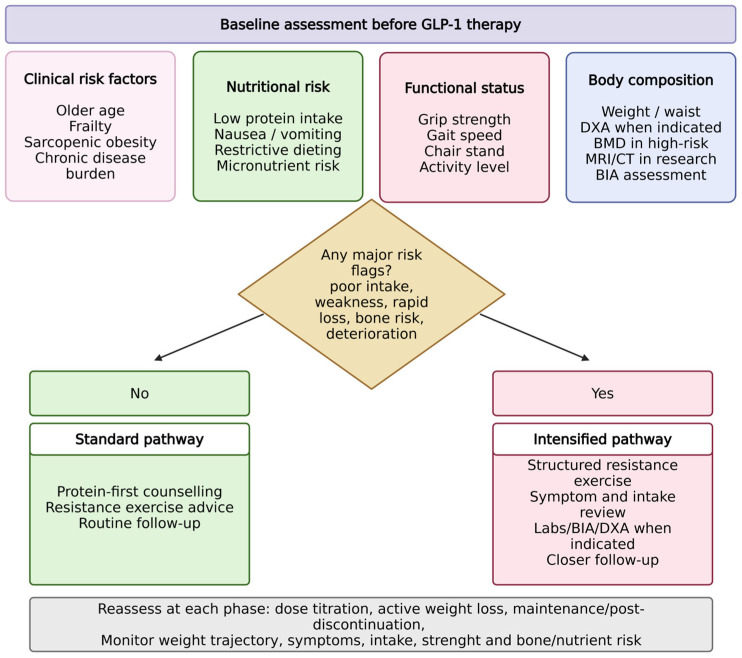
Pragmatic risk-stratification pathway for lean mass and musculoskeletal preservation during GLP-1-based obesity therapy. Baseline assessment integrates four domains: clinical risk factors, nutritional risk, functional status, and body composition. In patients without major risk flags, a standard pathway including protein-first counseling, resistance exercise advice, and routine follow-up is appropriate. Major risk flags, including poor intake, weakness, rapid weight loss, bone risk, or clinical deterioration, should prompt an intensified pathway with structured resistance exercise, symptom and intake review, laboratory and/or body composition reassessment when indicated, and closer follow-up. Risk status should be reassessed during dose titration, active weight loss, maintenance, and after treatment discontinuation, as nutritional intake, symptom burden, strength, and bone or nutrient risk may change over time. This framework is intended as a pragmatic clinical monitoring aid and should not be interpreted as a validated prediction score. BIA, bioelectrical impedance analysis; BMD, bone mineral density; CT, computed tomography; DXA, dual-energy X-ray absorptiometry; GLP-1, glucagon-like peptide-1; MRI, magnetic resonance imaging. Figure created with BioRender.com.

Supportive priorities differ by treatment phase. During initiation and dose escalation, the priorities are symptom control, hydration, and safeguarding protein intake. During active weight loss, structured resistance exercise, adequate protein, and surveillance for nutrient inadequacy become central. During maintenance or after withdrawal, the dominant risks are disengagement from exercise, deterioration in diet quality, and weight regain, which is well documented after treatment discontinuation [5,127]. Figure 1 translates these phase-specific priorities into a pragmatic risk-stratified pathway for lean mass and musculoskeletal preservation during GLP-1-based obesity therapy. Baseline assessment across clinical, nutritional, functional, and body composition domains helps identify patients suitable for standard follow-up versus those requiring intensified monitoring because of poor intake, weakness, rapid weight loss, bone risk, or clinical deterioration.

## 11. Priorities for Future Research and Emerging Therapies

### 11.1. Move Beyond Body Weight and Total Lean Mass

The next generation of GLP-1 body composition research should stop treating body weight and total lean mass as sufficient endpoints. Future trials need parallel assessment of fat mass, appendicular lean tissue, muscle quality by MRI or CT where feasible, strength, physical performance, cardiorespiratory fitness, bone density, dietary intake, and nutrient deficiency markers [12,13,17]. Without that multidimensional approach, the field will continue to generate debates driven more by terminology than by clinically meaningful outcomes.

### 11.2. Test Protein and Exercise Strategies Directly in GLP-1-Treated Populations

A second priority is direct interventional evidence. Much of the current nutritional advice is reasonable but indirect, extrapolated from the older-adult protein literature, caloric restriction studies, bariatric practice, and musculoskeletal physiology [57,58,59,126]. What is still missing are adequately powered GLP-1-specific trials that compare protein targets, protein timing strategies, oral protein supplementation, and structured resistance exercise programs during active pharmacotherapy. The field also needs better information on adherence; it is not enough to prescribe 1.2–1.5 g/kg/day of protein or progressive resistance training if most patients cannot sustain those recommendations in real-world conditions.

### 11.3. Biomarker-Guided and Phenotype-Guided Care

A third priority is phenotype-guided care. Older adults, people with sarcopenic obesity, individuals with diabetes and low physical reserve, and patients with marked gastrointestinal intolerance are unlikely to require identical supportive care intensity [18,21,77]. Biomarkers may help here, but so might simpler clinical phenotyping: baseline strength, phase angle, MRI-defined muscle quality, dietary pattern, symptom burden, and early change in protein intake. Omics studies in incretin-treated patients are still sparse, yet early metabolomic and proteomic findings suggest that treatment response could eventually be stratified beyond body weight change alone [106,107].

Prospective protocols are beginning to move in this direction. For example, an ongoing semaglutide study in older adults who are overweight and present insulin resistance is designed to assess physical function, body composition, and biomarkers of aging together rather than relying on weight alone [128]. This kind of design is more likely to answer clinically useful questions than another efficacy trial that reports large mean weight loss without clarifying whether musculoskeletal health improved in parallel.

### 11.4. Muscle-Preserving Combination Therapies

Combination therapy is another major frontier. The phase 2 BELIEVE trial showed that bimagrumab plus semaglutide can amplify fat loss while helping preserve lean mass relative to semaglutide alone, providing proof of concept for a muscle-preserving obesity pharmacotherapy strategy [129]. Meanwhile, newer incretin-based agents and combinations such as cagrilintide-semaglutide and retatrutide are pushing efficacy even further [130,131]. As these therapies mature, body composition, function, bone, and nutrient adequacy endpoints should be built into development programs rather than added as late substudies.

### 11.5. Standardize Outcomes and Reporting

Finally, the field needs better standardization. Published studies variably report lean body mass, fat-free mass, lean soft tissue, appendicular lean mass, muscle volume, muscle composition, or handgrip strength, often without sufficient explanation of what changed and why. Standard outcome sets should explicitly distinguish tissue quantity from tissue quality and include adverse nutritional outcomes as well as efficacy outcomes [16,17,27]. Clear terminology and reporting standards will be essential to avoid conflating any decline in lean mass with drug-induced sarcopenia.

## 12. Conclusions

GLP-1-based obesity pharmacotherapy generally improves body composition by preferentially reducing fat mass, but preservation of lean mass, muscle quality, function, bone, and nutritional adequacy is not automatic [14,36,37,39]. The most important conceptual correction is that DXA-derived lean mass loss is not synonymous with clinically significant muscle loss or sarcopenia [13,18,19].

The strongest current supportive care model is built around baseline nutritional and functional assessment, adequate protein intake, structured resistance exercise, management of gastrointestinal adverse effects, and risk-based monitoring for micronutrient inadequacy [16,17,21,55,76]. Oral protein support and selected supplements may help in some patients, but supplement-specific evidence remains uneven and should not be overstated. The field now needs GLP-1-specific trials that integrate imaging, functional testing, nutritional assessment, and omics so that preservation of lean mass is managed as a central component of high-quality obesity care rather than as an afterthought (summarized in Table 3). The main clinical implications of this review are summarized as concise take-home messages in Table 4.

## Figures and Tables

**Table 1:** Comparative evidence on lean mass loss and muscle deterioration during GLP-1 therapy.

Study/Method	Lean Signal	Fat Comparator	Quality/Function	Clinical Deterioration?
STEP 1 semaglutide/DXA [1,36]	LBM −9.7%	FM −19.3%; VF −27.4%; relative LBM +3.0 pp	Not assessed	No evidence
SURMOUNT-1 tirzepatide/DXA [2,37]	LST −5.6 kg; 26% of WL	FM −15.9 kg; 74% of WL	Not assessed	Possible only in high-risk phenotypes
SURPASS-3 MRI substudy [11]	Not reported	Liver/abdominal fat lower	Not assessed	Not assessable
SURPASS-3 post hoc MRI [39]	Muscle volume lower	Not central endpoint	Muscle fat infiltration improved	No simple deterioration
Liraglutide + exercise [23,24,25]	Not primary endpoint	Weight-loss maintenance	Fitness/BMD better with exercise	Risk is modifiable
Liraglutide thigh MRI [40]	Lower-limb tissue lower	Body-size reduction	Muscle fat/adverse composition lower	No deterioration signal
SEMALEAN semaglutide [26]	Lean mass modestly lower	Fat mass markedly lower	Handgrip strength improved	No functional deterioration
Meta-analyses/real-world [14,15,51]	Lean/FFM minority of WL; FFM 29.8%/24.8%	Fat loss predominant	Variable across methods	Overall no; monitor high-risk patients

Abbreviations: BMD, bone mineral density; DXA, dual-energy X-ray absorptiometry; FFM, fat-free mass; FM, fat mass; LBM, lean body mass; LST, lean soft tissue; MRI, magnetic resonance imaging; pp, percentage points; VF, visceral fat; WL, weight loss.

**Table 2:** Strength-of-evidence summary for nutrition, exercise, and supplementation strategies relevant to lean mass preservation during GLP-1-based obesity pharmacotherapy.

Intervention/Adjunct	Intended Goal	Key Supporting Evidence	Strength of Evidence	Practical Role
Protein-focused diet	Preserve lean mass and nutritional adequacy	Consensus guidance; limited GLP-1 dietary data; weight-loss RCT meta-analysis [16,17,55,79]	Moderate indirect; limited direct	First-line strategy; assess during titration
Oral protein support/meal replacements	Meet protein and nutrient targets when intake is low	No GLP-1 RCTs; indirect obesity and older-adult evidence [55,79,94,95]	Limited to moderate indirect	Use only when food intake is insufficient
Progressive resistance exercise	Preserve strength, function, bone, and lean mass quality	Liraglutide–exercise RCTs; broader obesity exercise evidence [23,24,25,53,56,87]	Moderate indirect; limited to moderate direct	Core co-intervention, especially in high-risk patients
Micronutrient monitoring and correction	Prevent or correct deficiency during low intake or GI intolerance	GLP-1 observational data; clinical nutrition guidance [55,76,77,78,91,92]	Limited direct observational	Risk-based; deficiency-directed
Creatine monohydrate	Support strength and training adaptation	No GLP-1 trials; older-adult resistance training evidence [97,98,99]	Limited indirect	Optional adjunct with resistance training
Leucine/EAA/HMB	Support muscle anabolism in catabolic or low-intake phenotypes	No semaglutide/tirzepatide trials; older-adult/HMB evidence [100,101,102]	Limited indirect	Selected high-risk patients only
Fiber/prebiotics/probiotics/synbiotics	Improve constipation, GI tolerance, or diet quality	Low fiber intake and GI symptoms reported; lean mass evidence absent [55,81,82,91,92]	Limited for symptoms; very limited for lean mass	Symptom-directed; not muscle-preserving therapy
Omega-3/multi-ingredient formulations	Provide anti-inflammatory or multimodal support	Mechanistic rationale; inconsistent clinical effects; no GLP-1 validation [95,103,104,105]	Very limited to limited	Research-priority or selected adjunct

Abbreviations: EAA, essential amino acids; GI, gastrointestinal; GLP-1, glucagon-like peptide-1; HMB, beta-hydroxy-beta-methylbutyrate; RCT, randomized controlled trial. Evidence strength reflects directness to GLP-1-treated populations, study design, consistency, and clinical applicability. Operationally, “moderate indirect” was used for interventions with consistent non-GLP-1 evidence applicable to weight-loss care, such as protein-focused diet and resistance exercise; “limited direct” for sparse GLP-1-specific observational or exploratory data, such as micronutrient monitoring; “limited indirect” for adjuncts supported mainly in the older-adult, sarcopenia, or exercise literature, such as creatine and leucine/EAA/HMB; and “very limited” for strategies without prospective GLP-1 validation or direct lean mass outcomes, such as probiotics for lean mass preservation and omics-guided personalization. This narrative classification is intended to improve interpretability and should not be considered a formal GRADE assessment.

**Table 3:** Clinical readiness of biomarkers and phenotyping tools for lean mass preservation during GLP-1-based obesity pharmacotherapy.

Biomarker/Tool	Readiness	Main Use	Key Limitation	References
Routine nutritional biomarkers: iron status, vitamin B12, folate, thiamine, 25-hydroxyvitamin D, calcium-related measures	Clinical use	Detect deficiency risk	Not muscle-specific	[76,78,109,119]
Routine metabolic and safety labs: renal function, glycemic markers, liver enzymes, lipids	Clinical use	Monitor safety and comorbidity	Not lean mass biomarkers	[16,17,76,78]
BIA	Clinical use	Fat mass, fat-free mass, hydration, and phase angle	Fat-free mass ≠ skeletal muscle, hydration- and equation-dependent	[31,32,33,34,115,116,117]
DXA	Clinical use/selected use	Fat mass, appendicular lean soft tissue, BMD	Lean mass ≠ skeletal muscle	[12,13,27,35]
MRI/CT muscle assessment	Emerging/selected use	Muscle volume and fat infiltration	Cost, access, CT radiation	[38,39]
D3-creatine dilution	Emerging/research use	Total body skeletal muscle mass	Not validated in GLP-1 trials	[41]
Creatinine:cystatin C indices	Emerging	Sarcopenia-risk enrichment	Moderate accuracy only	[118]
CRP, IL-6, TNF-alpha	Emerging/limited clinical value	Inflammatory context	Nonspecific	[108,109,119,120]
Myostatin, GDF-15	Research-only	Muscle/catabolic phenotyping	No GLP-1 thresholds	[121,122,123,124]
Metabolomics, proteomics, lipidomics, pharmacogenomics	Research-only	Mechanism and responder phenotyping	No clinical care pathway	[106,107,122,125]

Clinical-use markers may support routine nutritional or safety monitoring when interpreted in clinical context. Emerging tools may aid risk stratification or trial enrichment but are not validated as stand-alone clinical measures. Research-only markers should not guide routine GLP-1 supportive care. Abbreviations: BIA, bioelectrical impedance analysis; BMD, bone mineral density; CRP, C-reactive protein; CT, computed tomography; DXA, dual-energy X-ray absorptiometry; GDF-15, growth differentiation factor-15; GLP-1, glucagon-like peptide-1; IL-6, interleukin-6; MRI, magnetic resonance imaging; TNF-alpha, tumor necrosis factor-alpha.

**Table 4:** Key clinical take-home messages for lean-mass and musculoskeletal preservation during GLP-1-based obesity pharmacotherapy.

Clinical Issue	Take-Home Message
Lean mass loss	Do not equate DXA lean mass or BIA fat-free mass loss with sarcopenia without functional decline.
Body composition	Semaglutide and tirzepatide generally produce fat-dominant weight loss, but lean tissue loss may matter in high-risk patients.
Core care	Prioritize protein adequacy, resistance exercise, gastrointestinal symptom control, and risk-based micronutrient monitoring.
Intensified monitoring	Poor intake, rapid weight loss, weakness, mobility decline, bone risk, or suspected deficiency should prompt closer follow-up.
Emerging tools	Most supplements and omics-based personalization remain adjunctive or investigational, not routine standards of care.

Abbreviations: BIA, bioelectrical impedance analysis; DXA, dual-energy X-ray absorptiometry.

## Data Availability

No new data were created or analyzed in this study. Data sharing is not applicable to this article.

## Abbreviations

The following abbreviations are used in this manuscript:

ATP	adenosine triphosphate
BIA	bioelectrical impedance analysis
BMD	bone mineral density
B12	vitamin B12/cobalamin
CRP	C-reactive protein
CT	computed tomography
D3-creatine dilution	deuterated creatine dilution method
DRI	Dietary Reference Intake
DXA	dual-energy X-ray absorptiometry
GDF-15	growth differentiation factor-15
GIP	glucose-dependent insulinotropic polypeptide
GIP/GLP-1	glucose-dependent insulinotropic polypeptide/glucagon like peptide-1
GIPR	glucose-dependent insulinotropic polypeptide receptor
GLP-1	glucagon-like peptide-1
GLP1R	glucagon-like peptide-1 receptor
HMB	beta-hydroxy-beta-methylbutyrate
IGF-1	insulin-like growth factor-1
IL-6	interleukin-6
JAMA	Journal of the American Medical Association
MRI	magnetic resonance imaging
TNF-alpha/TNF-α	tumor necrosis factor-alpha
